# Facing the environment: onset and development of UV markings in young fish

**DOI:** 10.1038/srep13193

**Published:** 2015-08-18

**Authors:** Monica Gagliano, Martial Depczynski, Ulrike E. Siebeck

**Affiliations:** 1 Centre for Evolutionary Biology, School of Animal Biology, University of Western Australia, Crawley, WA 6009, Australia; 2 Australian Institute of Marine Science, University of Western Australia, Crawley, Western Australia 6009, Australia; 3 Oceans Institute, University of Western Australia, Crawley, WA, Australia; 4 School of Biomedical Sciences, University of Queensland, St Lucia, QLD 4072, Australia

## Abstract

Most colour patterns in animals represent an elegant compromise between conspicuousness to ensure effective communication with preferred receivers and camouflage to avoid attracting the attention of unwanted predators. Many species, including several coral reef fishes, overcome this conflict by using ultraviolet (UV) colouration and signalling, as these colours are visible only over short distances and are often invisible to their predators. Despite a great interest in their behavioural significance and ecological influence on survival, little is known about when these colours first develop on the bodies of free-living animals. Here we show for the first time that the UV facial patterns of a coral reef fish do not develop in captivity but only when juveniles experience the socio-behavioural conditions of their natural environment. Using field and laboratory experiments, we determined that the onset and early development of these UV facial markings did not occur at metamorphosis. Instead, juveniles developed the UV markings during their first two weeks on the reef. Exposure to different reef environments revealed significant plasticity in the development of these markings. The direct or indirect (through intraspecific interactions) exposure to predators is a likely candidate trigger for the plastic development of these UV markings in the wild.

The question of how animals produce some of the most spectacular visual displays with their colours and what they communicate with them has been challenging naturalists and philosophers alike since the time of Aristotle^1^. While there are several quite elaborate functional theories to explain animal coloration (for example, see^2^), ultimately and at the most basic level, colours and colour patterns are used to hide from the view of some while being seen and recognized by others. In a number of animal groups including amphibians, reptiles and fishes, the optimal balance between these two apparently conflicting conditions (i.e. crypsis in a predator-prey context *vs* conspicuousness in intraspecific communication) may be reached through changes in structural colouration^3^ such as blue, purple, ultraviolet and iridescent colours. Although fundamental questions about the development of coloured traits, including structural coloration have been tackled by various molecular, cellular and genetic laboratory studies (for example, see^4^,^5^), virtually nothing is known of their development under natural ecological settings. This is surprising given that these colours and colour patterns mediate environmental and social influences on behaviour and therefore, they represent an important form of morphological adaptation to ecological pressures^6^.

To understand how animals use structural colours and the behavioural and evolutionary implications of different strategies, it is important to elucidate how and when these colours and colour patterns develop in the wild and what information these visual signals encode and transmit as individuals go about their life searching for food and choosing mates while avoiding predators. As mentioned previously, the detectability of signals individuals use to exchange information is under strong selective pressure as greater detectability by an intended receiver brings about the potential for increased detectability by one’s own enemy^7^,^8^,^9^. In this regard, the use of ultraviolet (UV) signals is advantageous in allowing individuals to engage in short-range communication with conspecifics without drawing the attention of more distant predators. This is particularly true in aquatic environments, where UV signals are quickly attenuated in water and effectively disappear with distance (e.g. less than 5 m^10^,^11^;) and predators are generally UV-blind^12^,^13^. For such transfer of information among conspecifics to take place successfully, it is an essential prerequisite that both sender and receiver first develop and recognise an effective signalling system. It is well established that many invertebrate and vertebrate animals use UV signals for intraspecific communication^14^,^15^,^16^,^17^,^18^. Many coral reef fishes, for example, possess ultraviolet (UV) colour patterns^19^ and a variety of damselfish species, such as *Pomacentrus amboinensis*, display complex UV markings on their faces and fins^20^. Specifically, recent field-based behavioural experiments have convincingly revealed the ability of this damselfish to learn to discriminate fine-scale patterns like those seen on their faces^21^ and use this ability to distinguish conspecifics from heterospecifics^15^. Despite the behavioural significance of these UV facial markings to the ecology of this species, when these patterns first appear on the fish’s face and how their complexity develops over time has yet to be investigated.

The Ambon damselfish (*P. amboinensis*) is an abundant and common component of the fish community on the Great Barrier Reef, where it settles at the end of a pelagic larval phase to form reef-based, bottom dwelling social groups^22^. It has previously proven to be an ideal model species and system for examining ontogenetic changes in colouration^23^. It now offers a unique opportunity to investigate the development of UV colour patterns within a socio-behavioural and environmental context and more generally, to advance our understanding of animal signals and their relevance in animal social systems in the wild. This study therefore explored when in their life history, *P. amboinensis* first attain their UV colouration and whether the reef environment affects their early development.

Because UV markings in this species are associated with behavioural performance and survival^12^, we hypothesized that it would be most advantageous for these markings to develop rapidly and at settlement on the reef, a critical time when young damselfish are naïve to competitive interactions^24^ and predators^25^. Like in most damselfishes, the larval and juvenile-adult colouration in *P. amboinensis* are quite different and it is at the onset of metamorphosis (i.e. the end of the pelagic larval phase) that the bright and colourful juvenile-adult patterns emerge (i.e. within 6 hours of settling^26^;) from a mostly colourless larval body. We therefore hypothesized that if UV colouration is part of the structural and physiological changes associated with metamorphosis, the UV markings should appear concomitantly with all other colour markings at this stage.

In order to track the development of these markings, we conducted a series of laboratory and on-the-reef experiments where individuals were exposed to different ecological settings. Because UV signals in this species are important in mediating intraspecific interactions which often involve the sharing of resources^15^, we experimentally investigated whether food availability and social circumstances (i.e. presence/absence of conspecifics) affect the timing and development of these UV markings both in the lab and on the reef. Based on theoretical predictions^27^,^28^,^29^,^30^, we expected juveniles on food-supplemented reefs to forage more easily (i.e. less effort needed to search for food), take fewer risks and ultimately direct more energy to growth and development. We therefore hypothesized that food-supplemented juveniles would exhibit greater and more complex UV facial patterns than individuals on control reefs. Additionally, given that colour signals are known to vary even among individuals within a population, and that such variation may depend on factors such as age, condition or context^3^, we examined the level of variation in size and complexity of the UV markings in relation to fish size, age and condition. We expected the overall variation in the size and complexity to increase with age and body size among juveniles on both food-supplemented and control reefs. However, we hypothesized that variation in UV markings would be lower among juveniles living on food-supplemented reefs because food resources are abundant and the effort required to secure food would be more uniform among individuals. If this were the case, then these juveniles would be expected to allocate similar amounts of energy to growth, including the development of UV facial patterns.

## Results

### Differences in the onset of UV patterns among lab and reef treatments

**Figure 1:**
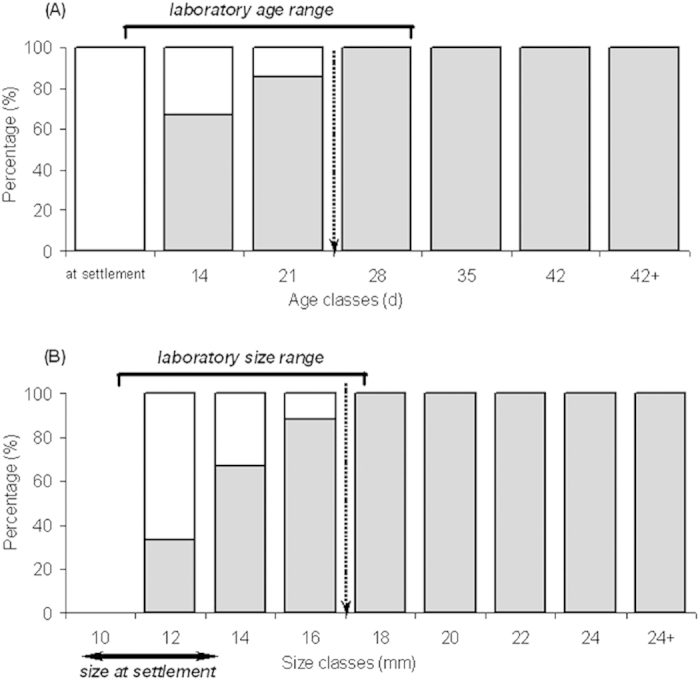
Percentage of wild *P. amboinensis* showing any UV reflectance. (**A**) None of the 68 recruits collected at the time of settlement possessed UV patterns. Nevertheless, the percentage of wild individuals exhibiting these patterns increased rapidly within days after settlement and over 85% of juveniles possessed them by the 3^rd^ week (marked by the dotted arrow). (**B**) Unexpectedly, same-size and bigger conspecifics maintained in the laboratory did not develop the UV patterns at all. Bars indicate wild individuals, where the relative percentage of individuals with and without UV facial marking is shown in grey and white respectively. The age and size ranges of fish maintained under laboratory conditions is shown by the solid line on top of the bars.

**Figure 2:**
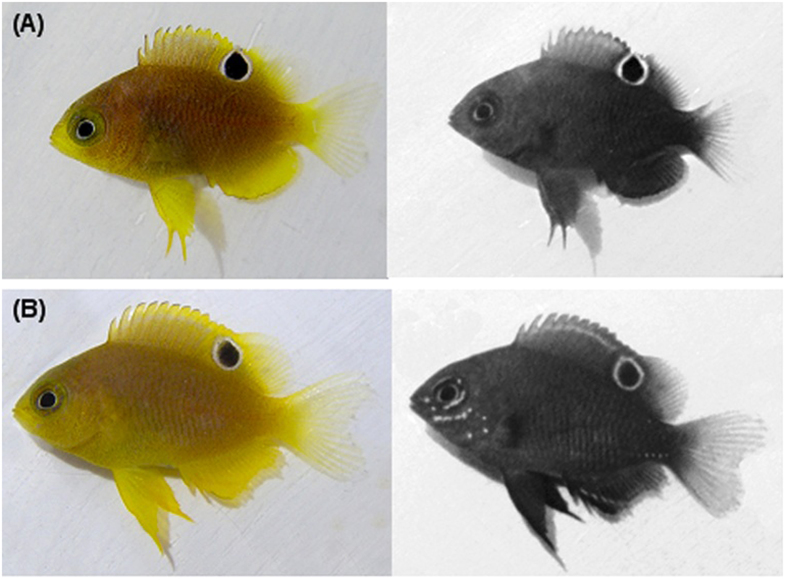
(**A**) Individual lacking UV patterns (SL = 12 mm, WT = 0.08 g, age 12 d) and (**B**) individual with UV patterns (SL = 15 mm, WT = 0.15 g, age 15.3 d), where UV-reflective areas appear white and UV-absorbing areas appear dark. Note – the white ring around the ocellus (i.e. eyespot on the posterior dorsal fin) and the iris both reflect UV.

We collected 68 *P. amboinensis* recruits in light traps at the time of settlement and none possessed UV patterns at this stage. Nevertheless on our patch reefs, the percentage of wild individuals exhibiting these patterns increased rapidly within days following settlement and almost 70% of individuals possessed well-developed UV patterns within 2 weeks of settlement and over 85% of wild juveniles possessed them by the 3^rd^ week (Figs 1a and 2). We found that irrespective of the experimental reef treatment individuals originated from, the onset of UV patterns in wild *P. amboinensis* was coupled with age and body size (Mann-Whitney test; age [pooled]: Z = 4.13, P < 0.001; size [pooled]: Z = 4.57, P < 0.001; Fig. 1) and independent of individual body condition (Z = 1.35, P = 0.18). Unexpectedly, same-age and older or same-size and bigger conspecifics maintained in the laboratory did not develop the UV patterns at all (Fig. 1a,b).

### Differences in the development of UV patterns among treatments

At the end of the 2-month experimental period, we found there was no difference in the number of juveniles with and without UV patterns that colonized food supplemented and control patch reefs (χ^2^ test, P = 1). Among individuals that developed UV patterns, fish living on control patch reefs exhibited a significantly larger UV-reflective area (mixed model ANOVAs; Total area: F_1,104 _= 5.39, P < 0.05; Eye area only: F_1,104 _= 6.16, P < 0.05; Facial area only: F_1,104 _= 4.06, P < 0.05) and a greater number of components in the eye pattern (mixed model ANOVA, F_1,104 _= 5.71, P < 0.05) compared to individuals from supplemented reefs.

### Differences in the variation of UV pattern among treatments

We found that variation (measured as CV) in the overall UV-reflective area was almost twice (1.96 times) as high among individuals on supplemented patch reefs as those on control reefs. Most of this variation was solely determined by differences in the facial portion of the UV pattern (i.e. supplemented reefs had 2.88 times more variability than control ones). In fact, variation in the eye portion of the UV pattern was much higher (2.42 times) among fish on control rather than supplemented reefs. Variation in the number of components in the eye and facial patterns separately or combined was similar between the 2 treatments.

### Size and age-related differences in UV pattern development among treatments

Regardless of the experimental treatment, older and larger individuals consistently developed both larger eye (SL [control]: R^2 ^= 0.34, r = 0.58, P < 0.001; SL [supplemented], R^2 ^= 0.35, r = 0.59, P < 0.001; Age [control]: R^2 ^= 0.35, r = 0.59 P < 0.001; Age [supplemented], R^2 ^= 0.33, r = 0.57, P < 0.001) and facial UV-reflective areas (SL [control]: R^2 ^= 0.54, r = 0.73, P < 0.001; SL [supplemented], R^2 ^= 0.57, r = 0.75, P < 0.001; Age [control]: R^2 ^= 0.45, r = 0.67, P < 0.001; Age [supplemented], R^2 ^= 0.52, r = 0.72, P < 0.001). Although there was no difference in mean body size (mixed model ANOVA, F_1,114 _= 0.09, P = 0.78), weight (mixed model ANOVA, F_1,114 _= 0.01, P = 0.93), condition (mixed model ANOVA, F_1,114 _= 0.01, P = 0.93) or age (mixed model ANOVA, F_1,101 _= 0.01, P = 0.92) between experimental reefs, control juveniles exhibited a considerably larger UV-reflective area at any given body size (ANCOVA, F_1,107 _= 9.26, P < 0.01), weight (ANCOVA, F_1,107 _= 9.50, P < 0.01), condition (ANCOVA, F_1,107 _= 5.04, P < 0.05) or age (ANCOVA, F_1,104 _= 10.38, P < 0.01) compared with supplemented individuals. Variation in body size, weight and condition was substantially greater among individuals living on supplemented reefs compared with fish from control reefs (5.83, 2.62 and 2.89 times respectively).

## Discussion

Molecular-genetic studies on the mechanisms of colour pattern formation have demonstrated how small differences in the regulation of structural genes may generate substantial changes in pattern elements and signal structure (e.g. eyespots in butterflies^31^; stripes formation in zebrafish^32^). While genes and the synthesis of specific proteins can specify pattern properties, we also know that, in insects, the onset of pattern ontogeny may be triggered by environmental circumstances (e.g. the aposematic pattern in the grasshopper *Schistocerca gregaria*^33^). To our knowledge, this is the first experimental study to investigate the onset and early development of UV patterns of a marine fish under natural settings. Findings from our field study combined with laboratory manipulations indicate that the development of these patterns is not associated with the process of metamorphosis as we hypothesized. We found that wild *P. amboinensis* recruits develop UV facial patterns within their first month on the reef, when all same-age conspecifics reared in our outdoor aquaria failed to do so despite otherwise metamorphosing and developing normally into the juvenile form. We can only conclude that the onset and development of these patterns is somehow linked to the exposure of an individual to its natural environment.

Neither food availability nor conspecific social settings affected the outcome of our outdoor aquaria manipulations, suggesting that neither factors directly trigger the onset or development of the UV patterns. Ultraviolet coloration in many animals, including fishes is produced structurally by layers of light-reflecting iridophores, which do not contain pigments of dietary origin^3^. We can therefore exclude that the lack of pattern development on the faces of our laboratory juveniles was due to an experimental artefact resulting from some dietary deficiency. Similarly, a comparison of juveniles who were maintained in conspecific groups in aquaria with wild juveniles who settled on a reef environment in the presence of conspecifics as well as other species indicates that even though the UV patterns in this species are brought into play in a social context^15^,^21^, the presence of conspecifics alone is insufficient to initiate their development.

So what is it about the natural reef environment that triggers the onset and development of these patterns in this species? Upon arrival onto reef habitats, tiny *P. amboinensis* recruits need to rapidly accomplish two vital tasks: eating and avoid being eaten. Quickly learning locally adaptive behaviours that allow rapid detection and evasive action to avoid capture is crucial for survival on the reef (>50% of individuals die within their first 5d on the reef^34^). Given that UV signalling enables individuals to communicate effectively without the associated danger of attracting the attention of predators^15^, the rapid development of UV facial markings as an operational ‘vocabulary’ should be favoured by selection. In this study, we found that the high number of *P. amboinensis* juveniles that developed these patterns within two weeks of settlement (~70%) matches well with the rapidly increasing survival rates recorded in this species by day 10 on the reef^35^. This supports our hypothesis that it would be most advantageous for these markings to develop rapidly and soon after settlement on the reef. Furthermore, although we did not specifically measure the link between survival and UV pattern development, our findings are consistent with previous observations suggesting that naïve *P. amboinensis* recruits that quickly develop into ‘reef-wise’ juveniles within their first two weeks on the reef have greater chances of surviving^36^.

The ability to exchange information such as potential predation threats with nearby conspecifics clearly offers major benefits, namely increased time for foraging activities as a result of a reduction in time spent watching out for predators (e.g. through collective detection^37^,^38^,^39^). However, because individuals must stay within close range to be able to spot conspecifics signalling danger^40^, the benefits of information transfer may be partially offset by increased density-dependent competition among conspecifics over food, which in coral reef fishes results in strong size hierarchies^41^. In our study, we found a greater size variation among individuals living on food-supplemented reefs compared to those on control reefs, suggesting that enhanced food availability probably resulted in the establishment of strong size hierarchies. As our daily food supplementation was provided as a nutritional pulse only, we acknowledge that this may have impacted on the natural spacing of individuals on the patch reefs and intensified competitive interactions among conspecifics by aggregating all individuals to a more concentrated food source. Still based on theoretical predictions^27^,^28^,^29^,^30^, we expected food-supplemented juveniles to forage more easily while taking fewer risks, hence able to allocate more energy to growth (including greater and more complex UV facial patterns) than individuals on control reefs. However, we detected no overall mean differences in body size, weight or condition between individuals from the two treatments, suggesting that differences in the spatial availability of food (i.e. concentrated vs natural) generated trade-offs with similar growth outcomes. Contrary to our expectations, we found that control individuals developed overall larger facial patterns than similar age/size fish whose diet was supplemented. We suggest that juveniles on control reefs experienced a more moderate level of competition, being forced by the naturally lower availability of food to distribute themselves further apart and venture further out into the water column to catch plankton. It has been previously demonstrated that this behaviour exposes individuals to a much greater risk of encountering predators, as most attacks occur on damselfish that are in the water column adjacent, rather than very close, to shelter^42^. Furthermore as shown in other animal systems, by being spaced further apart from each other, individuals may be under greater predation risk not only because they are an easier target^43^, but because they may be experiencing greater difficulty in spotting conspecifics signalling about predation threats^44^. Given that viewing distance affects the visibility of a signal^45^, the development of UV patterns of a larger size could enable juvenile *P. amboinensis* to retain their ability to send a signal that is sufficiently clear and detectable by conspecifics despite being further apart from each other.

The role that predator-prey interactions in the wild played in the formation of the UV patterns of *P. amboinensis* was not directly tested in our study; nonetheless our data provide some enticing support for the hypothesis that (direct and/or indirect) exposure to predators may be an important element. Clearly, the ability to detect one’s own enemies and consequently avoid potentially lethal confrontations is an exceptionally precious skill for individuals to possess. Ideally, young fish should have their antipredatory responses fully functional upon their first encounter with a predator without requiring any learning or prior experience^46^,^47^. Instead, they are generally born without the ability to even recognize their predators^48^. While some antipredatory behaviours may be innate, young and inexperienced individuals frequently develop and refine appropriate avoidance strategies over time through their own experience as well as through socially acquired information^49^. Whether UV signals in these juvenile fishes are used to transmit this kind of information or coordinate antipredatory behaviour and group cohesiveness (see^50^ on shoaling behaviour) is an interesting possibility, but it remains so far untested.

Overall, our results emphasize the importance of experience with the reef community to the development of fish UV coloration early in life. Specifically, this is the first study to provide direct empirical evidence that these UV facial markings develop only when individuals are exposed to their natural ecological and behavioural settings. Our results illustrate that competitive/cooperative interactions and risk-taking behaviours likely shape the developmental trajectories of these UV patterns in the wild, as larger patterns developed in juveniles that were more likely to venture further out into the water column and away from conspecifics. Given the social communication function of these UV facial markings among adults, we suggest that UV patterns in juveniles mediate the transfer of critical information among conspecifics about prevailing environmental conditions (e.g. potential predation risks) as well as species identity (e.g. if species and individual identity is visible over greater distances, it may help reduce aggressive interactions with neighbours^51^). Overall, the results support the idea that these UV markings and their plastic development early in life represent an exquisite form of morphological adaptation to the complexity of ecological and socio-behavioural pressures these young animals experience in nature.

## Methods

### (a) Model system

The focus of this study was the Ambon damselfish (*Pomacentrus amboinensis*), a common and abundant tropical fish on the fringing reefs around Lizard Island, Northern Great Barrier Reef. Following a dispersive larval life of 15–23 d, this species returns to the reef where it rapidly (<6 h) metamorphoses into the juvenile form by attaining the bright yellow body coloration and a conspicuous black dorsal eyespot distinctive of the juvenile benthic life stage^26^. The Ambon damselfish *P. amboinensis* settles directly into adult coral reef habitats and remains strongly site-attached throughout the rest of its life^52^,^53^, providing an ideal opportunity for tracking the life history of individual fish in the wild. Most importantly, *P. amboinensis* is an excellent model for this study because it possesses UV vision due to UV-transparent ocular media and a UV sensitive cone type^18^,^54^ and certainly adults rely on individually distinct UV facial patterns to modulate aggressive interactions with conspecifics and heterospecifics^15^ and also for species recognition^21^. Animal care and all experimental procedures were conducted in accordance with and approved by the James Cook University Animal Ethics guidelines (Permit Number: A-1254) and the Great Barrier Reef Marine Park Authority.

### (b) Onset of UV facial markings

To investigate UV pattern development, new *P. amboinensis* recruits were collected by light traps (see^55^ for trap design) as they approached reef habitats from the open ocean and immediately transferred to the laboratory. A random sample of 68 individuals was then photographed to establish whether UV patterns are already present at settlement.

### (c) Development of UV facial markings in the wild

To determine whether the onset and development of UV colour patterns of *P. amboinensis* depend on their feeding conditions, we constructed six experimental patch reefs on sandy bottom, 20–40 m off the edge of the main reef in 2–4 m of water. Experimental reefs were composed of a mixture of rubble and live coral, resembling patch reefs this species uses as a natural part of its habitat. Patch reefs were positioned 15–20 m apart to ensure they remained independent from each other by preventing any exchange of juveniles between reefs; such movement is highly unlikely however, as *P. amboinensis* remain strongly site-attached throughout benthic life once settled^35^,^52^. All experimental patch reefs were numbered and then randomly coded as either ‘supplemented’ or ‘control’ (n = 3 per treatment). Over a subsequent 2-month period, *P. amboinensis* recruits were allowed to naturally colonize all patch reefs. The diet of individuals settled on ‘supplemented’ reefs was enhanced with ground pilchards and barramundi pellets (Formula 87510V7 with 50% crude protein, 12% crude fat and 2·5% crude fibres) for 10 min each day, while individuals on the remaining reefs fed on naturally available plankton (‘control’). All patch reefs were checked daily to ensure that their structural integrity remained in good conditions. At the end of the 2-month period, a subsample of 20 individuals from each patch reef was collected and individual fish were placed in a small UV-transparent aquarium (5 cm × 2 cm × 5 cm) filled with fresh seawater for photography. A white piece of Perspex was inserted into the aquarium behind the fish to create an even background. Photographs were taken under natural sunlight with a digital camera (Sony DSC-F707). UV pictures were taken through a UV-pass filter (Oriel #59875) in combination with an infrared-absorbing filter (Oriel #59152) which resulted in a combined transmission of 350–400 nm. Manual settings were used for UV-photography (exposure: 1/20′, aperture: F2.0). Fish from food supplemented and control reefs were photographed in batches over the course of a day (ca. 9am–3pm), alternating between the two treatments. Colour photographs were taken without the filters with the camera set to automatic exposure and aperture. All individuals were then euthanized by cold shock and measured (standard length, mm [SL]), weighed (wet weight, g [WT]) and their body condition estimated using Fulton’s K index (K = WT/SL^3^). Sagittal otoliths were removed from all individuals and thin transverse sections through the nucleus allowed to determine their post-settlement ages.

### (d) Development of UV facial markings under laboratory conditions

To investigate the extent to which social conditions affect variation in onset and development of UV colour patterns, while accounting for the possible confounding effect of resource availability, feeding and social conditions were manipulated together in a laboratory experiment. To do so, new *P. amboinensis* recruits were collected using light traps as they approached reef habitats and immediately transferred to the laboratory. A random sample of 96 individuals was randomly assigned to four experimental treatments: Group fed *ad lib* (G+), Single fed *ad lib* (S+), Group fed every 3^rd^ d (G-) and Single fed every 3^rd^ d (S-). Fish in the fed *ad lib* treatments received 24 h-old *Artemia* sp. nauplii three times every day, while fish in the other treatments were fed once only every 3^rd^ day. Individuals were housed in either 1 l aquaria (n = 1 fish/aquarium; Single treatments) or 3 l aquaria (n = 3 fish/aquarium; Group treatments), which were all made of non-transparent plastic to ensure individual treatments remained visually isolated from each other. All aquaria were kept in a flow through system and supplied with a constant flow of unfiltered seawater (0.37 ± 0.06 l h^−1^) and held outdoors to ensure that temperature (28 ± 0.4 °C), salinity (34 ± 0.1 ppt) and light regimes remained as similar as possible to the nearby natural environment. Aquaria were inspected daily and cleaned of algal growth. In the morning of day 8, a total of 16 aquaria were randomly subsampled (two aquaria from each Group treatment and six from each Single treatment; total n = 24 individuals). Individual fish were photographed, measured, weighed and their condition estimated and sagittal otoliths removed as described above. We repeated this subsampling procedure on days 15, 22 and 29.

### (e) Measuring UV patterns

**Figure 3:**
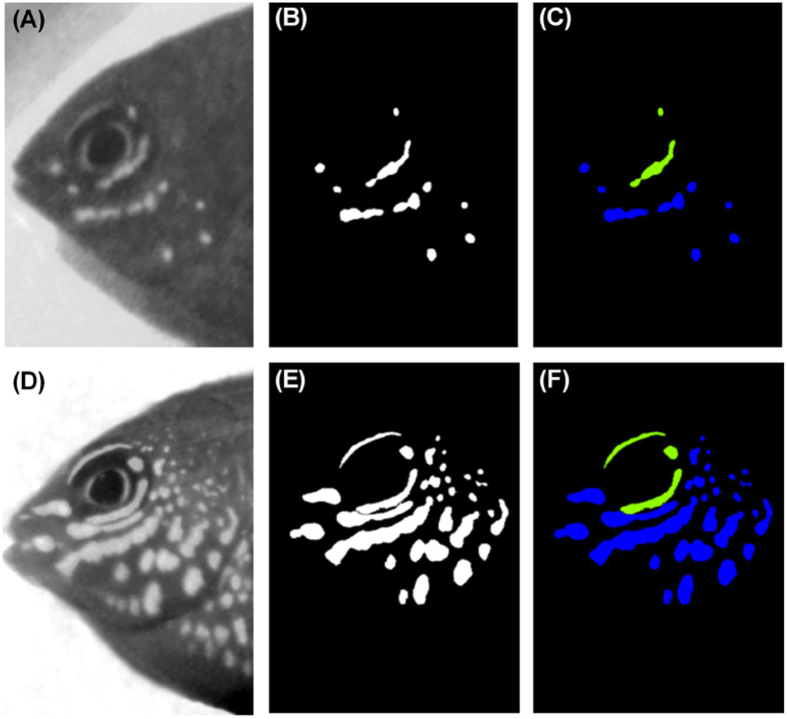
Images of fish faces were (**A,D**) converted into grey-scale images in which UV-reflective areas appeared lighter than UV-absorbing areas, and then (**B,E**) binarized in order to determine the number and size (in pixels) of white areas (pattern components). The eye and facial patterns were classified separately (green and blue areas respectively; **C,F**). The edge of the operculum was used to define the pattern boundary. Note – the inner ring around the iris was not included in the pattern analysis.

Images of fish faces were cropped (tip of nose to beginning of dorsal and pelvic fins), size standardized (500 × 700 pixels) and analysed using a program specifically developed for this task^56^. Briefly, images were converted into grey-scale images in which UV-reflective areas appeared lighter than UV-absorbing areas (Fig. 3A,D). A mask was used to separate the face of the fish from the background. Areas belonging to the UV-pattern could then be identified by setting a global threshold (130) within the grey-scale (0–255). Areas with pixel values below the threshold were identified as UV-absorbing while areas with pixel values above the threshold were identified as UV-reflective. Following this classification, the images were binarized (UV-reflective areas were set to 255 and UV-absorbing areas to 0, Fig. 3b,e) and the number and size (in pixels) of white areas (pattern components) was determined separately for the eye and facial pattern (Fig. 3c,f, green and blue areas respectively). The total number of components and total UV-reflective area were also calculated. Note that we did not attempt to quantify the strength of the UV signal, but were interested in the presence/absence of any UV reflective areas.

### (f) Light conditions in the laboratory and patch reefs

Irradiance was measured using a calibrated fibre-optic Spectrometer (USB2000, Ocean Optics, Florida USA) fitted with an irradiance probe and controlled by a palm computer. The system was taken underwater in a custom made underwater housing (Wills Housings, Melbourne, Australia) for irradiance measurements near the patch reefs. Results can be found as Supplementary Fig. S1 online.

## Additional Information

**How to cite this article**: Gagliano, M. *et al.* Facing the environment: onset and development of UV markings in young fish. *Sci. Rep.* **5**, 13193; doi: 10.1038/srep13193 (2015).

## Supplementary Material

Supplementary Information
