# Supplementary material for: Facing the environment: onset and development of UV markings in young fish
Source: Sci Rep. 2015 Aug 18;5:13193. doi: 10.1038/srep13193 (PMC4539538; doi:10.1038/srep13193)
Supplement: Supplementary Information [file srep13193-s1.pdf]

Supplementary Information

Facing the environment: onset and development of UV markings in young fish

Monica Gagliano[1*], Martial Depczynski[2] and Ulrike E. Siebeck[3]

[1] Centre for Evolutionary Biology, School of Animal Biology, University of Western Australia, Crawley, WA 6009, Australia

[2] Australian Institute of Marine Science, University of Western Australia, Crawley, Western Australia 6009, Australia

[3] School of Biomedical Sciences, University of Queensland, St Lucia, QLD 4072, Australia.

*To whom correspondence should be addressed. Email: monica.gagliano@uwa.edu.au

This PDF file includes:

Supplementary Figure 1. Light conditions in the laboratory and patch reefs

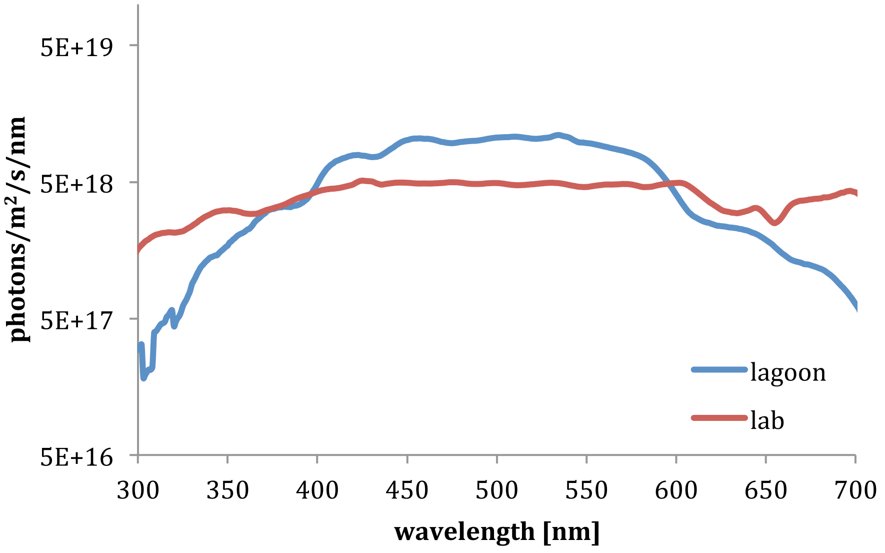

Figure S1. Light conditions in the laboratory and patch reefs. Irradiance levels (photons/m^2/s/nm) measured in the lab and near the patch reefs.
